# Participating in sports for individuals with autism spectrum disorder: shifting the focus from symptom relief to performance

**DOI:** 10.3389/fspor.2026.1800156

**Published:** 2026-07-01

**Authors:** Marte Fossflaten Tørring, Arve Vorland Pedersen

**Affiliations:** Department of Neuromedicine and Movement Science, Faculty of Medicine and Health Sciences, Norwegian University of Science and Technology, Trondheim, Norway

**Keywords:** coactive sports, community awareness, individual sports, interceptive actions, motor impairment, perception

## Abstract

In this hypothesis and theory paper, we propose a conceptual framework for identifying sports whose characteristics align with traits commonly associated with autism spectrum disorder (ASD). Inspired by reports in sports media about neurodiverse athletes who have excelled in various athletic activities, we hypothesized that some sports may especially suit individuals with ASD because they trigger fewer symptoms while also allowing them to play to their strengths. This article is not a review aimed at testing a hypothesis but instead presents our hypothesis, organizes relevant concepts, and discusses which sports may be best suited for athletes with ASD. To explore our hypothesis, we examined two recent reviews on sports-based interventions for individuals with ASD. Our examination revealed that most published studies have investigated symptom relief or the management of challenging behaviors, not participating in sports for enjoyment, to develop skills, or as a means of performance. Because few such studies have measured performance or the suitability of specific sports for individuals with ASD, we particularly considered whether some sports are especially suited for individuals with ASD for the purpose of not only competition but also performance. Following an overview of four sports categories, this article discusses how those categories are compatible with some common symptoms and features of ASD. Emphasizing the variability between and within the categories, we showcase certain sports in order to provide guidance for athletes and children with ASD, as well as their parents and therapists, in identifying appropriate sports for their particular forms of ASD.

## 1 Introduction

### 1.1 Autism spectrum disorder (ASD)

Children and adolescents with autism spectrum disorder (ASD) are not only less engaged in physical and leisure activities than their peers (1, 2) but also participate in sports less than their peers with or without disabilities (3). An explanation for these observed differences may lie in the core diagnostic features of ASD. According to the diagnostic criteria listed in the *DSM-5-TR* (4), *ASD* is defined by two criteria: (1) persistent difficulties in the social use of verbal and nonverbal communication and (2) restricted repetitive patterns of behavior. For a diagnosis of ASD, Criterion 1 needs to manifest in deficits in three domains: (i) social-emotional reciprocity, (ii) nonverbal communication, and (iii) developing and maintaining relationships. By contrast, Criterion 2 needs to manifest in at least two of four traits: (i) stereotypy and/or repetitive movements, (ii) inflexibility and/or ritualization, (iii) restricted and fixated interests, or (iv) hyper- or hypo-reactivity to sensory input. Those symptoms have to manifest during early development, not be explained by any intellectual disability, and be clinically significant in social and occupational functioning.

Although not a criterion of ASD, impaired motor function is a symptom associated with the disorder (5–8). Children with ASD, often described as clumsy and uncoordinated, typically show better manual dexterity than ball-handling skills. Common findings include impairments in postural control and in reach-to-grasp movements (5, 6), while other impairments include poor object control, poor object locomotion, and difficulties with using perception–action coupling strategies and with initiating and planning movements (7). However, those impairments may also overlap with related conditions such as attention deficit hyperactivity disorder (ADHD) and developmental coordination disorder (7–9).

Whether ASD-related differences in motor function reflect delays or deficits continues to be debated. Staples and Reid (10), for instance, have argued that insufficient practice may contribute to the differences because motor impairments in children with ASD cannot be explained by developmental delay only. Meanwhile, Gandotra et al. (7) have argued that social impairments among individuals with ASD hinder their participation in physical activity and therefore their development of motor skills. Individuals with ASD may indeed experience discomfort during physical activity and reduced self-esteem, and those challenges are often reinforced in childhood by limited adaptations and communication in physical education (11). However, despite motor delay and/or motor impairments, research has also shown that individuals with ASD can considerably improve their motor skills and perform well in certain sports (12, 13).

### 1.2 ASD and participating in sports

In sports media, stories abound about individuals with ASD who excel in sports such as ice hockey, track and field, and martial arts (14). More recently, scientific studies have even included elite athletes from different sports with ASD or other neurodivergent diagnoses (15–17). To account for those trends, the National Autistic Society (18) highlights that some traits of individuals with ASD may benefit their athletic performance, including advanced problem-solving skills, enjoyment with solving puzzles (14, 18), and excessive focus on a single task, usually with exceptional consistency and accuracy (18). Indeed, those features are shared between individuals with ASD and top athletes. Beyond that, the preference for routines, repetitive behavior, and strict schedules among individuals with ASD (4, 19) may enable them to excel in athletic performance.

Despite such potential strengths, the scientific literature rarely acknowledges that individuals with neurodivergent conditions, usually ADHD, can thrive in competitive sports. Of the handful of studies that have specifically examined athletes with ASD (16, 17), only one (15) has investigated high-performing athletes with the disorder. Even so, evidence suggests that many individuals with ASD could be especially motivated to participate in competitive sports that reward their usual behavior and perhaps demand less direct social interaction from them (16, 17, 20, 21). If individuals with ASD were to engage in sports that recast their traits not as setbacks but as assets, then their motivation to participate would not be imposed but intrinsic. Allowed to leverage their strengths as such, they might freely engage in physical activity that boosts their health and, in turn, experience the accompanying benefits on their social and cognitive behavior (17, 21).

### 1.3 Aim of our study

The aim of this hypothesis and theory paper is to explore whether certain sports may be especially compatible with ASD-associated traits and whether scientific studies on sports involving individuals with ASD, regardless of those studies' specific aims, support that hypothesis. We should note that although participating in sports to maximize performance may chiefly pertain to individuals with ASD who have fewer support-related needs, an awareness of differences in demands between sports is always important when introducing sports to individuals with ASD.

### 1.4 Conceptual approach

The scope of our study conducted for this hypothesis and theory paper was to propose and discuss, but not test, our hypothesis that the choice of sport matters if the performance of individuals with ASD is to be optimized. For that reason, we did not conduct an original search of the literature to identify relevant studies but instead used two recent, extensive reviews of scientific studies in which sports have been used as interventions for individuals with ASD (22, 23). We located the two reviews by searching several databases (i.e., Google Scholar, PubMed, and SPORTDiscus) for reviews including the words “sport(s)” and “autism” (or “ASD”). We sorted the findings by recency and chose the mentioned reviews because, together, they included well over 100 unique articles and were thus arguably representative of the current literature on the topic. Because our objective was not to evaluate the therapeutic effects of the examined sports, we do not discuss or present the results of the studies in the reviews. Instead, we wanted to discover trends, if any, existing between the sports chosen in studies involving individuals with ASD and their outcomes. We also explored whether the researchers had chosen sports based on an assumed alignment between their characteristics and ASD-associated symptoms and features and whether they had attributed their findings to those characteristics. Both aims helped us to gather support for our hypothesis that some sports are especially suited for skilled performance by individuals with ASD.

## 2 Discussion

### 2.1 Individuals with ASD and participating in sports according to the scientific literature

As discussed, research on sports in relation to individuals with ASD has focused on participating in sports as a means to increase physical activity, offer leisure, and/or manage or even improve symptoms of ASD, not as a context for athletic performance or achievement (22, 23). As a consequence, few studies have examined how the specific behaviors of individuals with ASD can benefit their sports performance, as Wertheim and Apstein (14) have similarly indicated. Few such studies have also included sport-specific performance measures or motor skill variables, and most have focused on individuals with greater support-related needs, usually very young children.

Despite the limited performance-oriented research on the topic, some notable trends are evident in the literature that we reviewed. Martial arts have frequently been examined, likely due to their emphasis on self-awareness and self-control and the repetitive structure of movements. By contrast, team sports have sometimes been described as overly complex for many individuals with ASD (12, 24). For example, Barak et al. (12), who reported results of a soccer-based intervention have suggested that although individuals with ASD are fully capable of learning simple soccer skills (e.g., shooting), they might struggle with learning complex, multistep skills (e.g., ball control and passing). Several authors have additionally argued that individual sports such as surfing (25–27) and skateboarding (28, 29) may be especially suitable for individuals with ASD, because those activities lack direct opponents and require limited social interaction.

In many articles examining martial arts interventions for individuals with ASD, the characteristics of the sports themselves are discussed as potential explanations for the positive outcomes observed, including improvements in social and/or communication skills. In the case of martial arts, such positive outcomes have been attributed to the inward focus and one-on-one interaction with instructors in those sports, along with the fact that martial arts are individual sports and socialization does not influence the results (13, 30–35). Along those lines, judo may be particularly suitable for individuals with ASD given its focus on self-regulation, control, discipline, and self-defense instead of combative techniques (36–38).

Studies employing interventions in basketball, meanwhile, have suggested that the sport's repetitive behavior may positively impact performance, measured as successful shooting rates, given relatively low variability in execution among athletes with ASD (39). Meanwhile, Jones et al. (40), who studied ice hockey, have discussed whether their results—improved hockey skills in children with ASD—were due to the one-on-one coaching strategies used instead of peer-focused strategies emphasizing social gains. Because children with ASD tend to become overwhelmed by sensory stimuli and consequently suffer from social isolation and limited communication skills, solitary sports such as surfing and running have also been proposed as good fits (25–27). Those sports require repetitive actions as well as demand determination and stamina—all traits that most children with ASD possess. Furthermore, those sports can be performed independently and without complicated rules or close contact with others.

However, the standout article, by Thomas et al., reports a case study in which an 11-year-old boy with ASD practiced skateboarding (29). Among the results, he not only acquired skateboarding skills but also transferred those skills across different skateboarding environments. As his performance improved, he became integrated into the local skateboarding community, and peers even asked him to teach them the techniques that he had mastered. In a subsequent study, the same authors demonstrated that another boy with ASD was similarly able to teach skateboarding skills to his two siblings (28). Although skateboarding is often considered to be a solitary recreational activity that does not involve teams or any cooperative goal-oriented behavior, children nevertheless often ride skateboards together in groups while performing tricks for one another. In that light, the sport may have considerable effects on their social skills.

### 2.2 Categorizing sports according to their general and specific demands on athletes

Because different sports place different general and specific demands on athletes in various ways, they also impose different demands across different individuals and groups. To discuss those various demands, in this section we establish a few meaningful categories of such demands in order to assess whether certain categories of sports may be especially or especially not suited to individuals with ASD. The categories presented are neither exhaustive nor mutually exclusive by any means; they simply seemed the most appropriate for providing various points of departure for our discussion.

One way to categorize sports is by the types of skills that they require. McMorris (41) has categorized sports skills along Gentile's (42) continuum of open and closed skills. Whereas an *open skill* involves a changing environment and a moving object, a *closed skill* is used in a static environment with a static object. That categorization is somewhat analogous to the frequently used distinction of complex (i.e., open) vs. simple (i.e., closed) motor skills (41).

To that continuum, Mackenzie (43) adds another dimension: the timing of skills. By extension, the timing of skills can be divided into *externally paced skills*—typically, open skills in which the pacing is directed by environmental factors such as opponents—and *self-paced skills*—typically, closed skills in which the athlete controls the pacing. Sports exemplifying those categories of skills are ball games, which require open, externally paced skills, and discus, which require closed, self-paced skills. For an additional factor of sports skills, Mackenzie describes whether the sports require a high or low degree of organization, which consequently affects the skill's attention-related factor: the need for attention during performance (e.g., in ball games) vs. performing more automatized, less attention-demanding skills (e.g., in running). The final factor that Mackenzie considers is the level of interaction with others while performing the skill, a category subdivided into *individual skills*, which are used in isolation; *coactive skills*, which are practiced simultaneously with others but involve no confrontation; and *interactive skills*, in which performance depends directly on others' performance.

Sports can be categorized not only by the type of skill required but also as either team or individual sports. By definition, *team sports* are all sports that involve two or more players competing together to achieve a goal against at least one other team (44). Due to involving several dimensions, team sports are generally characterized as complex sports (i.e., needing open skills), which generally demand skills in tactical, technical, and physical dimensions (e.g., a high level of attention) that determine the given sport's complexity. Team sports can be further differentiated according to whether their results require interaction with teammates and opponents all competing together at once (i.e., degree of interaction and timing of skills) or are based on the cumulative performances of multiple individuals that together contribute to the team's total score (45).

With loose reference to the mentioned categorizations, we divide team sports into two categories: complex, dynamic team sports (i.e., involving interactive, externally paced, open skills and demanding a high degree of organization in a changing environment with moving objects) and coactive team sports, in which a combined result is determined by accumulating individual performances (i.e., involving open or closed coactive skills that are externally or self-paced and demand only a limited degree of organization) (45).

In contrast to team sports, *individual sports*, as the name suggests, are sports in which each athlete competes alone (44). Such sports vary in their interactivity with opponents and, in this article, are categorized as either coactive nonconfrontational individual sports or interactive individual sports involving a direct opponent. In coactive nonconfrontational individual sports, athletes compete side by side using largely closed skills; such sports are self-paced and often demand limited organizational skills. By comparison, interactive individual sports may include both a changing environment and object (i.e., requiring open skills), be both externally and self-paced, and demand some organizational skills in addition to interaction with opponents.

Table 1 presents those four categories of sports together with the various demands that they place on athletes and how they relate to symptoms of ASD.

**Table 1:** General demands in the selected sports in relation to symptoms of ASD.

General demands in the four sports categories	Relevant, possibly limiting symptoms of ASD (4–8, 18–19, 50, 50–53, 55)	Complex dynamic team sports (i.e., with teammates and opponents) (43–46)	Coactive team sports (i.e., in which a combined result is determined by accumulated individual performance) (43–46, 48)	Coactive (i.e., nonconfrontational) individual sports (26–30, 43–48)	Interactive individual sports (i.e., with a direct opponent) (43–47)
Degree of interaction: Teammates, opponents, and referees (48–50)	Impaired social interactions with peers, preference to be alone, and lack of empathy, shared focus, and engagement with others	Interactive	Individual and coactive	Coactive and individual	Interactive with a few people
Practice environment (24)	Preference to be alone, no understanding of social rewards, sensitive to change in environment.	Social	Mostly individual	ndividual	Individual in a social context
Communication	Impaired nonverbal and verbal communication skills	Verbal and nonverbal communication	Limited communication with team	No need for communication	Some degree of communication with opponent and referee
Personality traits (19, 51–53)	Restricted interests, repetitive behavior, reduced flexible thinking, reduced emotional regulation, and lack of empathy, shared focus, and engagement with others	Agreeableness, sociotropy	Agreeableness, conscientiousness, autonomy	Conscientiousness, autonomy	Conscientiousness, autonomy
Organization	Preference to be alone and focus on self, high organizational skills, and patterns and puzzles	In a social context, high organization (i.e., attention needed)	Self-affordances, low organization (i.e., automatic)	Self-affordances, low organization (i.e., automatic)	In a social context, medium degree of organization (i.e., some attention needed)
Complexity (i.e., open or closed skill) (44, 56)	Sensitive to change in environment, challenged with handling moving objects	Open skill: Dynamic environment and task	Toward closed skill (might have change in environment and task)	Closed skill with static environment and task	Toward open skill with dynamic environment and task
Timing (49, 54)	Reduced motor skills, impaired object control, impaired IA	Externally paced	Toward self-paced	Self-paced	Toward externally paced
Perception (7, 12, 49, 54, 57)	Impairments in perceptual motor skills	High demand	Moderate demand	Moderate demand	High demand
Interceptive actions (IA) (49, 54, 55)	Problems with IA and timing	High demand with defending and attacking IA	No demand	No demand	High demand in some sports in the category
Motor planning (7, 49, 55)	Problems with motor planning	High demand	Various demands	Low demand	High demand
Pattern recall skills in motor function (57, 58)	Limitations in motor preparation	High demand	No demand	No demand	High demand
Examples of sports		Handball, soccer, ice hockey	Relay running, team swimming, team bowling	Running, bowling, disc golf, alpine skiing	Tennis, martial arts

In the table, the green color is good matches, orange contain moderate matches, and red cells contain poor matches between symptoms of ASD and the demands of sports within the category. Superscript numbers indicate sources listed in the reference list.

### 2.3 Psychological and organizational demands in the selection of sports in relation to symptoms of ASD

#### 2.3.1 Degree of interaction and communication in practice environments and competitions

As Table 1 shows, coactive sports, whether team or individual sports, place relatively few demands on social skills and communication abilities, provided that they are nonconfrontational. Such sports require limited interaction with opponents and teammates and are thus less demanding for athletes who experience difficulties with social interaction, social perception, and/or verbal and nonverbal communication skills (46). In parallel, interactive individual sports, especially nonconfrontational ones (e.g., tennis), even if involving a direct opponent, require less social interaction or communication with others except to provide factual information or the occasional polite exchange. Complex dynamic team sports, by contrast, require constant communication with not only teammates but also opponents, along with the ability to understand, anticipate, and adapt to the actions of both teammates and opponents, all of which requires advanced social perception skills (47). However, considering all the social limitations usually associated with ASD and that individuals with ASD may have specific difficulties with interpersonal physical activity (48), the fewer people involved in the sport, the better for athletes with ASD.

The practice environment may also influence the performance of athletes with ASD. In interviews conducted by Wertheim and Apstein (14), top athletes emphasized both having the opportunity to practice independently and according to their personal schedules as being advantages. That finding strengthens our argument that participating in coactive sports can benefit athletes with ASD.

#### 2.3.2 Personality characteristics

In team sports, athletes usually demonstrate relatively high levels of agreeableness and sociotropy (49). Of the two, agreeableness depends on interpersonal trust and relations, which demands caring about how others perform. Thus, for some individuals with ASD, participating in complex dynamic team sports may conflict with symptoms such as limited empathy, a lack of shared focus and interest in social rewards, and poor emotional regulation (19, 50, 51). However, those same symptoms and features may positively impact performance in coactive sports, which prioritize individual achievement and require relatively high levels of conscientiousness and autonomy and thus encourage personal distance from others and striving for individual excellence (49). In such sports, athletes with ASD may therefore have an advantage. The ability to imagine a sequence of events and perform that exact sequence without being disturbed by external factors is another advantage in some coactive sports (19).

#### 2.3.3 Organization and complexity

*Organization* refers to the level of attention needed in a skill or sport. Arguably, coactive sports require less organization than complex dynamic team sports and interactive individual sports, both of which require a relatively high degree of interaction, have numerous individuals present in the practice environment, and demand communication skills. In particular, most complex dynamic team sports require open skills, present an environment and tasks that are constantly changing, and need athletes to be flexible and focused outside themselves, all of which may be challenging for athletes with ASD. The two somewhat different types of coactive sports can demand both open and closed skills, such that certain sports in those categories might particularly suit athletes with ASD—for example, bowling, which involves competing at a constant temperature and in an unchanging environment (i.e., a bowling alley), compared with swimming, especially open-water swimming (e.g., in triathlons), which can entail waves and fluctuations in temperature. Added to that, the complexity of handling a tennis ball differs from the complexity of handling an opponent in martial arts. Thus, the individual athlete's ability to handle complexity needs to be evaluated against the specific demands of the sport in each particular category when considering which sport to pursue.

In complex dynamic team sports, the teammates, opponents, and referees all influence the performance of the athletes, and the athletes influence all of them as well. Those dynamics require a shared focus, social affordances, and social and communicational engagement. The opponents add complexity and unpredictability by directly engaging in sabotaging the performance of others by, for instance, tackling them or stealing a ball. The number of teammates and opponents also influences the complexity of the team dynamics.

#### 2.3.4 Timing, perception, and interceptive actions

A major component of perceptual motor skills is the ability to anticipate the future outcome of another's actions or the future position of an object in motion, as well as how and when to act in order to achieve a goal (52). In sports, athletes have to consider, as well as meet the demands of, temporal and spatial constraints when performing interceptive actions, which require moving the body, either entirely or in part, to be in the right place at the right time. Findings by Whyatt and Craig (53) showing poor ball skills and core balance among individuals with ASD suggest a problem with coordinating spatial and temporal aspects of movements. Individuals with ASD may also struggle with perceptual considerations before initiating movements (7, 53), including predicting where a ball will end up and how to move one's hand there in time to catch it. Simply put, the athlete needs to be aware of, adapt to, and respond to the ever-changing opportunities for action that each new situation affords (47, 52). If the individual with ASD has impairments in locomotion, temporal and spatial timing, and object control, then they may struggle to perform well in types of sports that require such perceptual skills (7, 13, 48). As indicated in Table 1, both complex dynamic team sports and interactive individual sports, due to their perception–action demands, are likely too demanding for most individuals with ASD.

Ball games entail complex actions that depend on timing and anticipatory skills. By the same token, reduced participation in ball games and poor skills in such games typically derive from poor visual–motor integration. Thus, sports involving a ball whose direction and speed athletes cannot entirely control (e.g., European handball and soccer) are likely not conducive to individuals with ASD, who typically struggle with those skills. Difficulties with motor planning and coordination might further hinder their performance in ball games. Ament et al. (54) found, for example, that children with ASD have impairments in motor skills when the skills require coupling visual and temporal feedback—for instance, when catching a ball.

These challenges can also be understood from a perceptual perspective, particularly in terms of affordances. Gibson (55) defines *affordances* as functional perceptions that an animal—in our case, humans—acts upon in relation to substances, surfaces, places, objects, and events in their environment. In sports, a specific action cannot be planned ahead in detail but has to be coordinated according to the situation and adjusted to the dynamic constraints, all of which suggests that a perceptual component is ever-present in all sports (56).

As Table 1 shows, both complex dynamic team sports and interactive individual sports demand interaction. As such, they offer perceptual affordances that athletes need to be able to detect and use. Affordances in an interactive context include not only the individual athlete but also other players on the team, the team as a whole, and the players on the opposing team or, in the case of individual sports, the opponent (47). In complex dynamic team sports, the sport consists of a triadic attacker–defender–goal system in which the athlete has to make decisions based on the affordances found within the defender–goal dyad. The attacker's goal is to destabilize that dyad and thereby alter preexisting affordances and exploit the new affordances that emerge (56). For athletes with ASD, who may be self-focused, those perceptual affordances might be impossible to perceive, let alone comprehend.

### 2.4 Physical demands in the selection of sports in relation to features of ASD

Table 1 highlights some features of sports that place organizational, coordinative, and perceptual demands on athletes and thus may be limiting for individuals with ASD. However, sports place physical demands on athletes as well. Although we have described impairments in motor functions among individuals with ASD, other evidence suggests that those functions can be improved with training (10, 12, 13). According to Ohrberg (57), though some children with ASD may be unable to participate at certain competitive levels, adjusting the age group or competition level may allow their meaningful participation (p. 55). That point, together with Wertheim and Apstein's (14) argument that highly skilled performance is attainable in various sports, suggest that sports in all four categories have to be considered when identifying ideal sports for individuals with ASD. To aid that effort, Table 2 presents details to consider when choosing sports for individuals with ASD. Two sports from each category, with somewhat different demands for athletes, have been chosen to illustrate the difference between and within the categories. The table and the following sections also highlight previously mentioned differences and demands within each category across specific sports.

**Table 2:** Physical demands in the selection of sports in relation to symptoms of ASD.

Physical demands	Relevant, possibly limiting symptoms of ASD (4–10, 50, 55–56)	Complex dynamic team sports (i.e., with teammates and opponents)		Coactive team sports (i.e., in which a combined result is determined by accumulated individual performance)		Coactive (i.e., nonconfrontational) individual sports		Interactive individual sports (i.e., with a direct opponent)	
		Handball (49, 65)	Ice hockey (60–64)	Relay running (66)	Team bowling(67)	Alpine skiing (68–71)	Disc golf (72)	Tennis (73)	Martial arts (12, 31–36, 58, 74–75)
Anaerobic power	Reduced gross motor skills, hypotonia, motor apraxia, toe-walking	High	High	High	Low	High	Low	Moderate	High
Aerobic fitness	Reduced gross motor skills, hypotonia, toe-walking, motor apraxia	High	High	High	Low	High	Low	High	High
Agility	Clumsiness, motor apraxia, difficulties with motor planning	High	High	Low	Low	High	Low	High	High
Strength	Reduced muscle tone	High	High	Moderate	Low	High	Low	Moderate	High
Flexibility	Reduced flexibility	Moderate	High	Low	Low	High	Low	Low	High
Balance	Reduced core balance ability	Moderate	High	Low	Low	High	Low	Moderate	High
Gross motor skills	Apraxia, reduced gross motor skills	Moderate	Moderate	Low	Low	High	Low	Moderate	High
Fine motor skills	Reduced reach-to-grasp movement, apraxia, increased manual dexterity	Moderate	Moderate	Low	Low	Low	Low	Moderate	Moderate
External elements	Reduced ball skills, reduced reach-to-grasp movements, reduced object control	Ball	Stick, puck	Baton	Ball	Skis	Frisbee	Racket, ball	None
Coordination	Poor coordination, apraxia, clumsiness	High	High	Low	Low	High	Low	High	High

In the table, the green color is good matches, orange contain moderate matches, and red cells contain poor matches between symptoms of ASD and the demands of sports within the category. Superscript numbers indicate sources listed in the reference list.

#### 2.4.1 Complex dynamic team sports with teammates and opponents

In complex dynamic team sports, the immense demands on athletes' physical, technical, and tactical skills hardly suit individuals who experience difficulties with coordination, agility, and handling external elements (Table 2). As mentioned, individuals with ASD may have relatively poor ball skills and difficulties with performing interceptive actions, which would make most complex dynamic team sports seem altogether too demanding. Even so, considerable variability also exists along the continuum of ASD as well as within sports within the category. For example, Wertheim and Apstein (14) have described the high-level performance of an athlete with ASD as a goalkeeper in ice hockey. That position is perhaps somewhat comparable with the category of coactive team sports and would thus be recommended if athletes wished to participate in a complex dynamic team sport*.* In ice hockey, a goalkeeper needs to be quick and explosive, albeit only occasionally and across a limited space, because they are in or near the goal crease throughout each game (58–60). Beyond that, the position of goalkeeper demands flexibility and repetitive movements instead of puck- and stickhandling (58). Although ice hockey is indeed a team sport, no player is as isolated throughout the game as the goalkeepers (61, 62). Goalkeepers can also practice independently and need to follow position-specific rules during games (59, 60). Although interceptive actions are required, goalkeepers additionally have minimal involvement in passing and limited social interaction, which makes the role comparatively accessible for athletes with ASD.

By contrast, goalkeepers in other sports, including handball and soccer, engage more frequently with teammates, have to recognize opportunities, and rely heavily on anticipatory and pattern-recall skills (47, 63). Furthermore, motor planning and reaction, which demand spatial and temporal timing, are pivotal. Those circumstances make the position demanding for athletes with ASD and relatively similar to outfield positions in complex dynamic team sports.

#### 2.4.2 Coactive team sports

Relay running, appearing within the recommended sports category in both Tables 1, 2, is indeed recommended for individuals with ASD because motor impairments in the disorder may not be especially limiting to performance. The features described as being demanding in Table 2 are understood in relation to motor function impairments described by Ming et al. (5), including apraxia, hypotonia, toe-walking, and generally reduced gross motor function. Although all those features limit the ability to run, Ming et al. (5) have also emphasized that because adults usually lack the mentioned impairments, athletic performance can be improved greatly with training. The only interactive and communication element would be the baton handoff, which in contemporary relay races might be called a “blind handoff” because communication is unnecessary (64).

Bowling and team bowling are two other coactive sports that may be among the most suitable for athletes with ASD. Arguably, bowling's physical demands are limited and thus achievable for most athletes with ASD. In team bowling, teammates take turns bowling, and physical and communicational collaboration between teammates may be rather limited. Because the goal in bowling is to bowl the ball down the lane and topple all 10 pins in every attempt (65), the ball is indeed an external element, but there are no interceptive actions. Moreover, the sport has few task-related and environmental constraints, all of which are consistent, and its features of detail orientation and the perfectibility of a solution are also advantageous for athletes with ASD. Not only can the sport's consistency, repetitive behavior, and rigidity enable highly skilled performance (14, 18), but practicing can also be done independently, which might minimize the impact of social difficulties and reduced sensitivity to one's environment. Although competitions involve noise and other people, such disturbances can be eliminated, at least in part, by the athlete's ability to self-focus, or else the athlete can use noise-canceling devices because auditory information is unnecessary for performance.

#### 2.4.3 Coactive nonconfrontational individual sports

In alpine skiing, as an exemplar of coactive nonconfrontational individual sports, performance depends on muscular strength, anaerobic power, anaerobic endurance, aerobic endurance, coordination, agility, balance, and flexibility (66, 67). Although those physical and physiological features are typically demanding for athletes with ASD, they are also highly trainable (10, 12, 13). Alpine skiing is complex because success relies on the athlete–environment interaction, which requires sustained focus on the task at hand and precise technical execution (68, 69). In that light, the strong self-focus of athletes with ASD may improve their ability to perform in alpine skiing; however, due to substantial physical demands, highly skilled performance may be difficult (18, 19).

Albeit remarkably different, another coactive sport is disc golf, a relative new sport whose aim is to land a disc in a mounted basket at a distance in the fewest throws possible. Following a concept similar to traditional golf, a disc golf course usually has 9 to 18 holes, each with a par score (i.e., the preset number of throws per hole needed to earn 0 points, as is desired). The Disc Golf Association (70) claims that the sport is suitable for everyone who can throw a disc, regardless of their fitness level. That claim is verified in Table 2, which shows that disc golf is clearly a sport that would be recommended to athletes with ASD.

#### 2.4.4 Interactive individual sports involving a direct opponent

At first glance, tennis might seem to be too physically demanding for most athletes with ASD. The aim is to hit the ball over the net so that the opponent, on the other side of the net, cannot hit it back across (71). The sport has elements of social affordances and interceptive actions, as well as a host of additional demands (Tables 1, 2). Nevertheless, tennis may be a good match for athletes with ASD because it has limited task-related and environmental constraints and is traditionally played in relative silence.

In martial arts, athletes need to perceive distance and target positions in order to select the proper comeback, which consequently constrains and continuously changes the affordances and further demands rapid body-scaled perception (56). As indicated in Table 2, the sport's physical demands are immense; however, the individual constraints (e.g., symptoms) of ASD might benefit athletes who participate in martial arts. Martial arts require focus during practice and competitions, such that athletes have to filter other perceptual information out from the environment and focus exclusively on themselves and their opponent (72). Although practice occurs in a social setting, neither communication nor social skills are necessary because the sport's focus is self-improvement, not teamwork (73). The strong self-focus observed among individuals with ASD may help them in martial arts. At the same time, the sport requires toughness, self-belief, motivation, and a tolerance for pain (72), which may put individuals with ASD, who generally have low self-esteem, at a disadvantage. However, because practice and goal attainment nurture self-belief and self-esteem, the athletes' self-esteem may increase during practice. As mentioned, martial arts are also often used as an intervention for problematic behavior in children and youth with ASD. Studies on the topic (13, 30, 32, 34, 35, 73–75) have revealed the positive effects of martial arts on behavior, and, by extension, examining whether the effects are also due to mastering the sport would be valuable.

## 3 Conclusion and implications

This article has described the severe lack of research on athletic performance among individuals with ASD and shown that published studies have focused on participating in sports as a means of symptom management and training in social skills, not on athletic performance. We argue that the specific demands of each sport being considered for individuals with ASD to engage in should be assessed according to the individual's unique limitations and assets, so that they can experience joy, mastery, and meaningfulness from participating, as everyone who engages in sports should.

Coactive sports seem particularly fitting for athletes with ASD, especially team bowling, disc golf, and relay running, in which both participation and performance are attainable. However, without any scientific research, those recommendations do not exactly answer the question of which sports individuals with ASD should pursue. Even so, it is relatively clear that such individuals could participate and perform, even excel, in sports that value repetitive behavior, inward focus, limited interaction with peers, and limited perceptual motor demands. At the same time, the variety along the continuum of ASD is so immense that it is impossible to identify a one-size-fits-all sport for individuals with ASD.

Future studies should systematically investigate performance-related outcomes across different categories of sports for individuals with ASD, particularly coactive sports, which appear promising based on theoretical fit but lack empirical validation. In addition, studies should examine training approaches, coaching strategies, and environmental adaptations that may enhance performance among athletes with ASD, including roles or positions (e.g., goalkeeper) that align with individual strengths. Last, greater attention to athletes' subjective experiences, through qualitative or mixed-methods designs, is essential to elucidate motivation, enjoyment, and long-term engagement in sport among individuals with ASD.

## Data Availability

The original contributions presented in the study are included in the article/Supplementary Material, further inquiries can be directed to the corresponding author.
